# Neutral and Stable Equilibria of Genetic Systems and the Hardy–Weinberg Principle: Limitations of the Chi-Square Test and Advantages of Auto-Correlation Functions of Allele Frequencies

**DOI:** 10.3389/fgene.2012.00276

**Published:** 2012-12-19

**Authors:** Francisco Bosco, Diogo Castro, Marcelo R. S. Briones

**Affiliations:** ^1^Departamento de Microbiologia, Imunologia e Parasitologia, Universidade Federal de São PauloSão Paulo, São Paulo, Brazil; ^2^Laboratório de Genômica Evolutiva e Biocomplexidade, Universidade Federal de São PauloSão Paulo, São Paulo, Brazil; ^3^Departamento de Medicina, Disciplina de Infectologia, Universidade Federal de São PauloSão Paulo, São Paulo, Brazil

**Keywords:** Hardy–Weinberg, allele frequencies, evolution, population genetics

## Abstract

Since the foundations of Population Genetics the notion of genetic equilibrium (in close analogy with Classical Mechanics) has been associated with the Hardy–Weinberg (HW) principle and the identification of equilibrium is currently assumed by stating that the HW axioms are valid if appropriate values of χ^2^ (*p* < 0.05) are observed in experiments. Here we show by numerical experiments with the genetic system of one locus/two alleles that considering large ensembles of populations the χ^2^-test is not decisive and may lead to false negatives in random mating populations and false positives in non-random mating populations. This result confirms the logical statement that statistical tests cannot be used to deduce if the genetic population is under the HW conditions. Furthermore, we show that under the HW conditions populations of any size evolve in time according to what can be identified as neutral dynamics to which the very notion of equilibrium is unattainable for any practical purpose. Therefore, under the HW conditions the identification of equilibrium properties needs a different approach and the use of more appropriate concepts. We also show that by relaxing the condition of random mating the dynamics acquires all the characteristics of asymptotic stable equilibrium. As a consequence our results show that the question of equilibrium in genetic systems should be approached in close analogy to non-equilibrium statistical physics and its observability should be focused on dynamical quantities like the typical decay properties of the allelic auto-correlation function in time. In this perspective one should abandon the classical notion of genetic equilibrium and its relation to the HW proportions and open investigations in the direction of searching for unifying general principles of population genetic transformations capable to take in consideration these systems in their full complexity.

## Introduction

In his letter to the Editor of Science Hardy (1908) showed that under specific conditions the simple two allele system (A, a) has the property that the allele frequencies (*p*, *q*) determine the genotype frequencies (AA, aa, Aa) obeying proportions given by the simple well-known relation *p*^2^ + *q*^2^ + 2*pq* = 1. Independently, the German physician Weinberg (1908) arrived to similar results. In the 1930s the synthetic theory proposed that under the Hardy–Weinberg (HW) conditions genetic systems attain an “equilibrium” state characterized by the genotype frequency proportions obtained directly from the allele frequencies as stated in the HW relation above. This definition of equilibrium in genetic systems became part of the well-known HW Principle (Hartl and Clarke, 2007) which formally states that: “If a genetic population is such that (1) organisms are diploid, (2) reproduction is sexual, (3) generations do not overlap, (4) mating is random, (5) the size of the population is significantly large, (6) allele frequencies are equal in the sexes, and (7) there is no migration, mutation, or selection, then the genotype frequencies in the population are given by weighted products of the allele frequencies. In the case of the one locus-two allele system the allele frequencies [(A, a) = (*p*, *q*)] give directly the genotype frequencies [(AA, aa, Aa) = (*p*^2^, *q*^2^, 2*pq*)].” Under the above conditions it is easy to demonstrate the following corollary: “For a population satisfying the HW conditions the allele frequencies are constant in time.” The notion of HW equilibrium adopted in the synthetic theory derives from the above corollary.

Attempting to establish a conceptual “bridge” with the definition of mechanical equilibrium in Newtonian mechanics, the synthetic theory adopts the idea that the existence of a time invariant observable leads to the hypothesis that the genetic population would be in equilibrium with no net “external forces” acting on the system (Stephens, 2004; Hartl and Clarke, 2007). From this simple reasoning it became broadly accepted that these external forces should be represented by selection and therefore the theory derives the well-known definition of evolution based on the variation of the allele frequencies. This vision of biological evolution is strongly supported by the well-defined concept of mechanical equilibrium of (classical) physical systems: if the sum of all external forces is null, the physical system is said to be isolated and its (macroscopic) mechanical state (well characterized by appropriate mechanical variables of its constituents) is unaltered. In this situation the system is said to be in mechanical equilibrium. This notion is a result of the combined use of the well-known first and second Newton’s principles.

In the case of genetic systems, assuming that selection “forces” are not acting on the population, the system would be free of external forces and would be in a “genetic state” for which the allele frequencies are constant in time. The idea of genetic equilibrium state related to zero “external forces” would follow immediately. Although this theoretical construct may be appealing it is important to note that it is no more than an analogy with serious difficulties to be formally established as it is done in classical mechanics: in classical mechanics the notion of a force acting on a system (as a result of fundamental physical interaction) has the formal status of a (operational) definition. In fact, from the formal point of view, the first two Newtonian Principles are definitions, and therefore cannot be proven: the first principle is equivalent to the definition of (inertial) mass and the second can be seen as a prescription to calculate the force acting on the system. The notion of mechanical equilibrium emerges from the first two principles in the following way: according to the second principle (the sum of all forces acting on the mechanical system is defined as *F* = *m*·*a*, where the force *F* is understood to be the cause of the transformation of the state of movement, necessarily external to the system and the acceleration is identified with the observable system’s response) if the resultant of all forces is null then the system should behave in time according to the first principle, namely driven by its own inertia. In this case we say the system is in a special mechanical state called equilibrium, which is a very appealing notion since according to the same first principle the system should then remain in this state (eternally) unless an external action takes the system out of its state of mechanical equilibrium. Therefore, the idea of mechanical equilibrium is related to the impossibility to change the mechanical state of the system by means of the system itself. In other words, the state of a mechanical system can only be altered by means of the interaction of the system of interest with another one. The interaction with another system is described by the third principle; therefore, the three principles contemplate both the definition and identification of equilibrium state (combined use of the first two principles) and how the interaction with an external agent take the system out of an equilibrium state (mediated by the third principle). As definitions of inertia and force, the first two principles are validated through results from specific applications. Only the third principle, the Action–Reaction Principle, has a more fundamental role since it is related to the (universal) law of conservation of momentum and intrinsic symmetries of the physical system. The formal results of classical mechanics cannot be derived solely by the first two principles. The third law is in the very foundation of the theory since it addresses the physical phenomenon itself (the interaction of two physical systems obeys basic principles).

In genetic systems viewed in analogy with classical mechanical systems, natural selection appears as the definition of a cause external to the system and capable of changing the system’s state (assumed to be defined by allelic frequencies), such that if natural selection is absent the system should evolve according to its own “inertia.” Therefore, if the analogy is fully considered, natural selection should be placed as a formal definition to be validated through concrete applications. In this line of thought, the biological law (or natural principle) analogous to the third law of classical mechanics is still to be found.

The above arguments should be sufficient to understand that the notion of genetic equilibrium in the framework of the synthetic theory is (at most) related to the concept of statistical equilibrium. Its relevance as a natural principle should be supported by experiments. In this respect it is interesting to note that in his original study, Hardy (1908) carefully chose the word “stability” and not equilibrium to describe the statistical invariance of allele frequencies. This is more than a semantics difference because a system can be stable without being (necessarily) at equilibrium, as for example is the case of metastable states in non-equilibrium thermodynamics and therefore stability and equilibrium do not necessarily refer to the same physical properties of dynamic systems (Kivelson and Reiss, 1999).

As it appears in textbooks on population genetics the canonical χ^2^ statistical test is currently used to compare observed and estimated proportions of alleles (Hartl and Clarke, 2007). The basic idea is that if the measured number of genotypes in the population is statistically close enough (χ^2^ < 3.8414… and correspondingly *p* < 0.05) to the theoretically expected (given by the HW proportions) then the population is said to be in HW equilibrium; in other words the χ^2^-test is used as an indicator of deviation from randomness. Nevertheless, as stated in the HW theorem the set of properties the population has to satisfy constitute necessary but not sufficient conditions for the invariance of the allele frequencies. Therefore, in rigorous terms by merely satisfying the statistical condition χ^2^ < 3.8414… one cannot guarantee random mating or any of the other conditions (or premises) of the HW principle. To prove this statement it suffices to find a counterexample, namely: in a genetic system for which at least one of the conditions of the HW theorem is violated it is possible to satisfy the condition χ^2^ < 3.8414… Analytical counterexamples and a possible generalization of the HW principle have already been studied by Li (1988) and Stark (1976, 1980, 2005, 2006a,b) considering the case of infinite populations under non-random mating.

The χ^2^-test is used in experimental trials with genetic systems as a tool with the specific goal to identify if the genetic system is in a state of equilibrium characterized (and defined) by the conditions stated in the HW principle. This strategy, normally adopted in many studies (Salanti et al., 2005; Rodriguez et al., 2009), would be logically correct if the HW theorem would state necessary and sufficient conditions. The conclusion that the genetic system is in a state of (HW) equilibrium because the measured χ^2^ < 3.8414… (or any other value) lacks logical foundation. In fact, its usefulness and limitations for the study of genetic systems are currently under investigation (Rohlfs and Weir, 2008; Engels, 2009).

To exploit more deeply the possibilities of the system through counterexamples and other statistical properties relevant for the basic concept of genetic equilibrium, we performed numerical experiments with the simplest genetic system of two alleles and three genotypes (AA, Aa, aa) for very large but finite populations. The numerical simulations show that the stable ensemble distribution of χ^2^ leads to inconclusiveness about random/non-random mating for populations of any finite size. The constancy of the allele frequencies can only be observed for rigorously infinite populations under strict HW conditions. As a consequence we present strong arguments and evidences supporting the conclusion that for any finite genetic population under the HW conditions the time evolution of the allele frequencies is dynamically neutral with a corresponding equilibrium state attainable only in the infinite time horizon, and therefore unattainable and not well characterized by isolated observations. On the other hand in the case of non-random mating the allele frequencies obeys a stable dynamics with a well characterized stable equilibrium state. As a result we propose that the problem of characterizing equilibrium states in genetic systems should be addressed in dynamical terms where the use of statistical quantities like χ^2^ would acquire a secondary (auxiliary and not conclusive) role. Therefore, in order to address the question of equilibrium states in genetic systems we should move toward a different perspective by looking the genetic population as a dynamic system whose main signatures are coded in the time series of useful observables. To make our point more clear we analyze the time evolution of the two alleles – one locus system through numerical simulations.

## Methods

**Figure 1:**
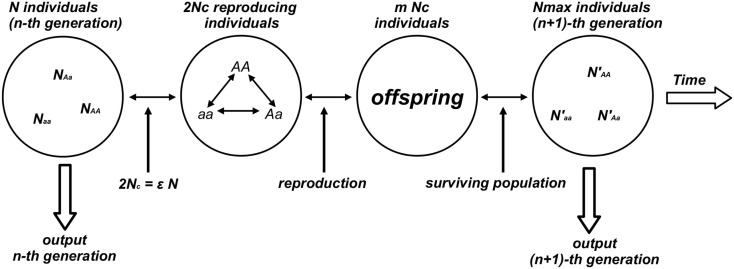
**Diagram of the numerical experiment that generates the time evolution of the genetic population**. This figure illustrates one reproductive cycle. From the *n*-th generation the algorithm randomly selects *N*_c_ couples (2*N*_c_ reproducing individuals) corresponding to a fraction ε of the total population *N*. Couples are selected according to specific rules (random or not) and reproduction takes place with a mean number *m* of descendants per couple. When the population grows at geometric rate (*m* > 2) a prescribed number *N*_max_ of individuals composes the surviving population defined as the (*n* + 1)-th generation.

Numerical experiments were performed for the genetic system defined by a population of *N* individuals composed by the three subpopulations with *N*_AA_, *N*_Aa_, *N*_aa_ individuals such that *N* = *N*_AA_ + *N*_Aa_ + *N*_aa_. At each time step *N*_c_ reproducing couples (2*N*_c_ = ε *N* surviving/reproducing individuals composing the effective population, 0 < ε < 1) were chosen from the population according to a prescribed probability distribution (*P*_AA_, *P*_Aa_, *P*_aa_) that assigns to an individual a stationary probability to be chosen among the individuals of the same genotype. Each individual is chosen according to the probability distribution (*P*_ij_) and two subsequently chosen individuals form one reproducing couple. This process continues till the total number of chosen individuals reaches the value 2*N*_c_ composing the reproductive population (or effective population). Reproduction gives birth to a new generation composed by *mN*_c_ individuals. To avoid the case of geometric explosion of the population size (*m* > 2) the model fixes an upper bound for the population size *N*_max_: if *mN*_c_ > *N*_max_ then the exceeding individuals [(*mN*_c_ − *N*_max_) individuals] are randomly chosen and eliminated from the population. Each generation step is completed when the couples reproduce giving birth to the next generation of *N*_max_ individuals. The generation step is considered as the discrete time unit of the model (see Figure 1).

The case of random mating corresponds to equiprobable individuals; the probabilities *P*_ij_ to choose any individual are approximately given by the instantaneous frequencies *P*_ij_ ≈ *f*_ij_ = *N*_ij_/*N* for each of the genotypes; in the case *N* → ∞ and *N*_ij_ → ∞ the fractions *f*_ij_ converge to the *a priori* probabilities *P*_ij_. For *N* → ∞ under the remaining HW conditions the frequencies *f*_ij_ are time invariant and relate to the allele frequencies through HW proportions. For each chosen couple the model specified the number of descendants that could be deterministic and equal for every couple or could be probabilistically chosen from the values 1, 2, 3, or 4. In this case the population had a mean and stable reproductive capacity. Clearly, if the mean reproductive capacity was *m* = 2 then the size of the population fluctuated around a constant mean value; if *m* > 2 the population size grows geometrically – here (after reproduction) the fixed number *N*_max_ of individuals are randomly chosen as viable ones (composing the next generation) in order to avoid the exponential explosion of the population size; if *m* < 2 the population trivially goes to extinction in finite time.

The numerical simulation starts by prescribing an initial population with *N* individuals with the genotype profile (*N*_AA_, *N*_Aa_, *N*_aa_) at (*t* = 0); the input parameters are the maximum population size *N*_max_, a set of four stationary probabilities assigned to the four possible values of the reproductive capacity of each couple (determining the mean reproductive capacity *m*), a probability matrix used to fix mating chances among the individuals (random mating being a special case), the fraction ε of surviving/reproducing individuals and the total number of generation steps (the simulation duration). It is important to note that during the process of mating the condition of random mating is strongly dependent on the number of (still) uncoupled individuals because when two individuals are chosen to form a couple they are removed from the population such that the next couple is chosen among a smaller number of individuals. Clearly this may introduce a sampling effect over the mating process. Therefore, in order to minimize these effects and guarantee homogeneous mating rules for all individuals, a fraction of the (already large) population has to be disregarded. For instance, random mating is assured as much as one imposes large values of the reproductive capacity together with large values of death rate. The parameter ε specifies the fraction of the populations that survive and reproduce; clearly this parameter has to be carefully chosen in combination with the parameter *m* in order to avoid extinction of the population during the numerical simulation.

As outputs we measured the genotype frequencies, allele frequencies, and the value of χ^2^ over the total population at each time step. The computational platform allows accessing the time series of those quantities or to fix one time step *t*_obs_ and observe the same quantities for a statistical ensemble of populations that evolve from *t* = 0 with the same initial conditions. In that way it is possible to study the statistical properties of the system over the system’s history or over the statistical (abstract) ensemble of populations for one fixed generation at time *t*_obs_. To favor clarity and avoid unnecessary complications in order to achieve our main objective to discuss the notion of equilibrium in genetic systems, we focus our attention on the effects of finite size of populations and random/non-random mating conditions.

The source program was written in C++ on a 64-bits Linux platform. The executable file is available upon request.

## Results

**Figure 2:**
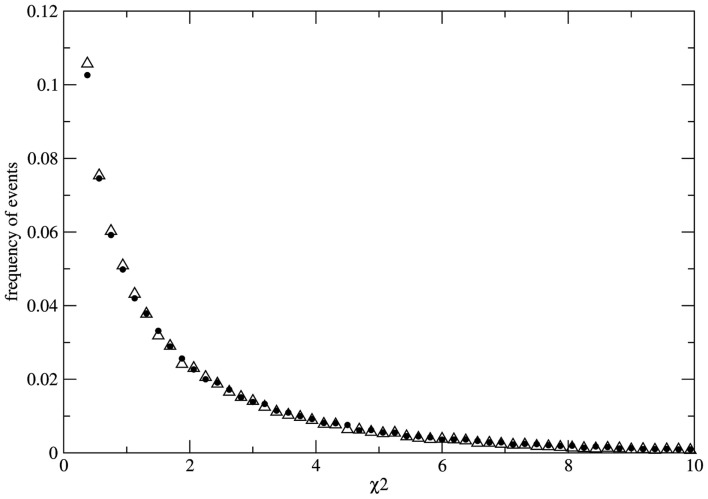
**Distributions *P*(χ2) over the statistical ensemble of 10^5^ populations composed by 3.0 × 10^6^ individuals each**. The values of χ^2^ are measured at fixed generation (simulation time unit) 30. The case of random mating (full dots) and the case of non-random mating (empty triangles) are well fitted by exponential distributions for χ^2^ > 0.4 with a small deviation from the exponential function for small χ^2^ values. Both distributions are very stable with respect to the size of the statistical ensemble. The distributions are used to evaluate the probability of false negative (for the case of random mating) and the probability of false positive (for the case of non-random mating).

To study the fluctuations of χ^2^ we avoid the problem of sampling by calculating the distribution of possible values of χ^2^ over a statistical ensemble *Z*_L_(*N*) made up of *L* copies of identical populations of size *N*. For each population of the ensemble we evolve the system till a fixed time point where the instantaneous value of χ^2^ is measured. As a result we obtain the *Z*_L_(*N*) – ensemble distribution of possible values of χ^2^ for populations of size *N*, at fixed time. As an example we present the results of numerical experiments for *N* = 3.0 × 10^6^ and *L* = 10^5^. Initially all populations of the ensemble are identical with the same genotype distribution *N*_aa_ = *N*_AA_ = *N*_Aa_ = 10 × 10^6^. At each time step the number of reproducing individuals is fixed at *N*_rep_ = 1.8 × 10^6^ in such a way that imbalance of the mating probabilities (due to sampling over the finite population) is minimized at each time step. Geometric reproduction rate is imposed to guarantee the same value of *N*_rep_ at each time step. The measurement of χ^2^ is made for each population of the ensemble of 10^5^ populations at generation 30. Here we present the results of two typical simulations. Figure 2 shows the ensemble distributions of χ^2^ for populations under random mating condition and one example of non-random mating condition where the mating probabilities are fixed as *P*(AA) = 0.23, *P*(aa) = 0.23, *P*(Aa) = 0.54 in order to choose reproducing couples (these values are chosen just for the sake of presentation; indistinguishable distributions can be also obtained for different values of the mating probabilities). As it can be clearly observed the two distributions are almost indistinguishable. As we consider finite populations the distribution of χ^2^ over the ensemble is not the (canonical) one

$$P\left(x\right)=\frac{\text{1}}{{\text{2}}^{k∕\text{2}}G\left(k∕\text{2}\right)}x^{k∕\text{2}-\text{1}}{\text{e}}^{-x∕\text{2}}$$

[where *G*(*m*) is the gamma function and *k* is the number of degrees of freedom; for the system of two alleles – one locus *k* = 1] applicable for the case of infinite populations. In fact, if the population is finite then the largest possible value of the variable χ^2^ is the population size *N* and therefore the appropriate distribution function $\chi_{N}^{\text{2}}$ -distribution should be null for χ^2^ > *N*. As a consequence for finite populations the critical value $\chi_{\text{c}}^{\text{2}}\approx \text{3}\text{.8414}$ corresponds to a *p*-value *p* > 0.05.

In the numerical experiment related to Figure 2 both distributions have a well fitted exponential tail for χ^2^ > 0.4. With the help of the ensemble distribution it is possible to estimate the probability of false negatives in the case of random mating as *P*(χ^2^ > 3.8414) ≈ 0.146 if we accept the critical value 3.8414… We note that this probability is slowly varying in the range *N* = 10^4^–10^6^ even if the statistical ensemble is sufficiently large. Moreover, it is very difficult to obtain the exact distribution for finite populations due to slow convergence with respect to the ensemble size. It is clear that the convergence to the χ^2^-distribution for infinite populations [*P*(χ^2^ > 3.8414) ≈ 0.05] is faster if we consider random samples over large populations. Nevertheless, the main conclusions presented here are unaltered by considering the χ^2^-distributions over the total population or over samples. As a result even if the population is known to be under the HW conditions there is always an irreducible probability of at least 0.05 (for the ideal case of inferences with respect to infinite populations) that the χ^2^ estimator leads to false negatives. More dramatic results are obtained for the case of non-random mating. The ensemble distribution *P*(χ^2^) is very similar to the case of random mating with a pronounced exponential tail and the probability of false positive can be estimated as *P*(χ^2^ < 3.8414) ≈ 0.848 [or *P*(χ^2^ < 3.8414) ≈ 0.95 in the case of the distribution over random samples]. Therefore if the population is subject to an external bias (not detectable by other means) resulting in non-random mating there is a significant probability to get measurements leading to false positives.

As a conclusion, the χ^2^ criterion can only be considered as a poor estimation of the conditions (HW or not) under which the genetic system is submitted. In other words, from the simple observation that $\chi^{\text{2}}<\chi_{\text{c}}^{\text{2}}$ for any critical value $\chi_{\text{c}}^{\text{2}}$ the existence or not of equilibrium states due to natural causes/mechanisms cannot be logically sustained.

The above numerical results put in evidence the intrinsic limitations for the use of statistical estimators to address fundamental properties of evolving genetic systems like equilibrium states and their nature. In fact, on fundamental grounds genetic systems are better characterized as physical systems subject to size fluctuations (finite size effects) and open in respect to energy and matter flow.

The strong limitations of statistical tests to identify and characterize equilibrium states of the genetic system, forces us to examine the concept of equilibrium in a deeper way by appealing to its dynamical aspects. In fact, the relevant aspect of characterizing equilibrium states relates to the very dynamical properties of the system that are inscribed in its time evolution.

When the concept of equilibrium is used as part of theoretical frameworks it becomes a crucial point to characterize the type(s) of equilibrium (equilibria) the natural system (or the model) has. In fact, the full characterization of the different equilibria as a function of the system’s parameters may be seen as a reliable portrait of the model encoding the very essence of the principles governing the time evolution of the system. A coarse classification defines three types of equilibria: stable, neutral, and unstable. The canonical way to identify the nature of the equilibrium state is to describe the system’s behavior close to (in a small neighborhood of) the equilibrium state by means of small perturbations. For instance, in simple mechanical systems the case of a small rigid ball sliding on a concave surface under the action of the gravitational field and a small friction force is a simple example of a system having a stable equilibrium state (the position where the ball has minimum potential energy). If the surface is convex the point of maximal potential energy is an unstable equilibrium state and in the case of a small ball on a flat and horizontal surface every point on the surface is a neutral equilibrium state of the mechanical system. Here the small ball constitutes the physical system and the surface is external to it and may be seen as the “environment.” The equilibrium state is an attribute of the system as a result of its interaction with its environment. In all three cases a fundamental property of equilibrium states is the time invariance under the action of the dynamic law; if *E* is the identified equilibrium state and *S* represents the dynamics then formally equilibrium states satisfy the relation *S*(*E*) = *E* meaning that equilibrium states are special states of the system which are invariant under the action of the dynamic law evolving the system.

For systems whose states are given by appropriate distribution functions of suitable observables the same concepts may be applied; one may talk about neutral, stable or, unstable equilibrium states that are essentially statistical as a result of the intrinsic stochastic nature of the system. A simple example of stable (collective) states is described by the distribution of velocities of molecules of a (non-reacting) gas in thermal equilibrium with a heat bath; small perturbations on the gas may shift the system out of its equilibrium state but the system spontaneously goes back to its initial (stable equilibrium) state.

For genetic systems under the HW conditions the allele frequencies are fixed from the initial point (*t* = 0) and remain constant for the rest of the future system’s history provided that the population is infinite. Small perturbations on the initial genotype frequencies will affect the time evolution by shifting the system to a new set of HW proportions which will remain constant in time with new values. This means that the genetic system under the HW conditions is better described in terms of neutral equilibrium states. In the appropriate jargon one should say “genetic systems under the HW conditions evolve according to neutral dynamics.” This neutral aspect of the dynamics is the property that allows genetic systems of finite size to evolve toward the fixation of alleles by random genetic drift.

**Figure 3:**
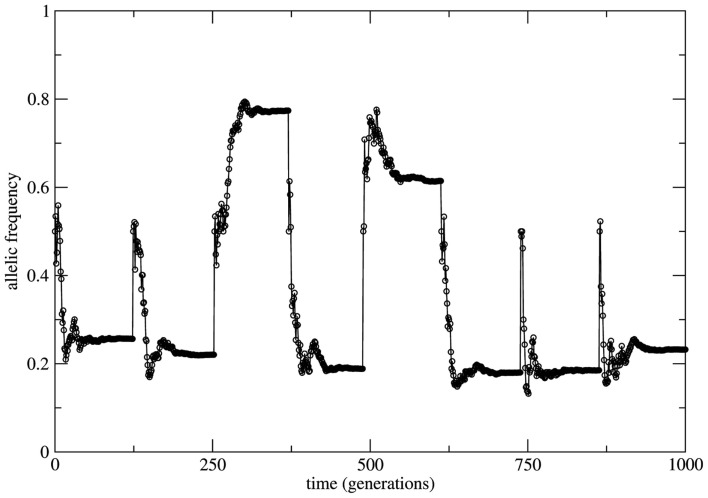
**Time series of the allele a frequency for the case of random mating**. To highlight the role of neutral dynamics an initial population with the profile *N*_aa_ = *N*_AA_ = 10 and *N*_Aa_ = 20 (allele frequency *f*_a_ = 0.5) grows till a fixed size of *N* = 3.0 × 3.0 × 10^7^ individuals. After reaching this maximal population size the allele frequency *f*_a_ reaches approximately constant values interrupted by subsequent imposed bottlenecks reducing the population to its initial profile. After each bottleneck event a new transient takes place leading to new (approximately constant) value of *f*_a_ due to the neutral characteristic of the dynamics.

If a genetic population is bounded in size (due to the balance between birth and death rates) and if the remaining HW conditions are respected, at each time step of observation (generation) the system is in a statistical state characterized by HW proportions. The change from one state to a new one is driven by the small fluctuations of the genotype frequencies which constitute a source of allele frequency changes internal to the system. Here the random mating condition is respected at each time step but mating probabilities for each genotype fluctuate along the system’s history together with the genotype frequencies. Neutrality is a very special property of equilibrium states. In fact, it is the limiting case between stability and instability. Moreover, under HW conditions the genetic system has an infinite (uncountable) number of neutral equilibrium states labeled by all the values 0 < *f*_a_ = 1 − *f*_A_ < 1 of the allele frequencies. This uncountable proliferation of neutral equilibria has a deep dynamical significance. It implies that the long term behavior of the system (measured in terms of the asymptotic time invariant values of the allele frequencies) may be as diverse as all the possible values 0 < *f*_a_ < 1. As a consequence this asymptotic state depends on the genotype frequencies at *t* = 0. In Figure 3 we show the allele frequency time series of a numerical experiment where the population grows geometrically till the value *N* = 3.0 × 10^7^ starting with the genotype profile *N*_AA_ = 10, *N*_Aa_ = 20, and *N*_aa_ = 10; random mating is considered at each time step.

The time necessary to reach the maximal population size is a transient period (around 100 generations) during which the population is subjected to fluctuations in the genotype frequencies. As the population grows these fluctuations decrease and the allele frequencies reach an approximate stationary value. Subsequent bottlenecks are imposed to the population bringing it to its initial configuration. As it can be easily observed the approximately stationary values of the allele frequency are different after each bottleneck; the asymptotic behavior of the allele frequencies depends on the transient. This is a direct consequence of the neutral character of dynamics of finite populations under the HW conditions.

**Figure 4:**
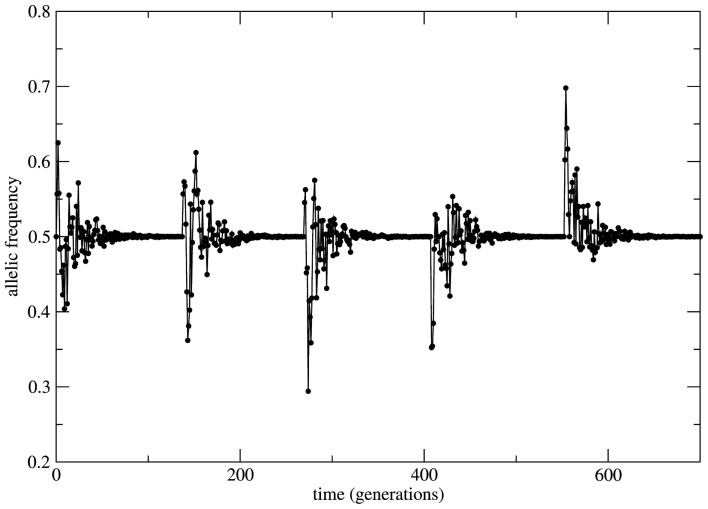
**Time series of the allele a frequency for the case of non-random mating**. The initial and the after bottleneck sizes of the population and its maximal value are the same as in the case of Figure 3. After each bottleneck event the population experiences a transient period of approximately 100 generations and then the allele frequency stabilizes at *f*_a_ = 0.5. The subsequent bottleneck events imposed to the population shows the robustness of the transient size and of the asymptotic value of the allele frequency due to stable character of the dynamics.

This scenario changes dramatically if the bounded population is conditioned by fixed *a priori* stable probabilities of mating relaxing the random mating HW condition. In this situation the system is driven by stable probabilities fixed by sources necessarily external to the system (note that from the logics of the model construction any information source out of the set of alleles (A, a) is formally considered as external to the system). The overall effect over the time behavior of allele frequencies is time invariance with stable properties. Now small perturbations on the genotype frequencies rapidly die out in time and the initial (unperturbed) equilibrium state is restored. As it can be seen in Figure 4 the initial small population (identical to the case of Figure 3) passes by a transient of approximately 100 generations and stabilizes its allele frequency around the asymptotic value *f*_a_ = 0.5. Just after stabilization of the allele frequency the population is subjected to a bottleneck reducing its size to its initial value; a new transient takes place and the allele frequency converges to the same previous asymptotic value *f*_a_ = 0.5. This convergence to the same value is the signature of stable dynamics.

It is important to note that typical values of χ^2^ are remarkably small if the equilibrium state of the system is either neutral or stable. Clearly under unbalanced mating probabilities the system becomes trapped on a stable state and fixation of alleles is impossible. The fact that the genetic system may evolve under neutral or stable dynamics (depending on the balanced or unbalanced nature of the mating probabilities) suggests the following plausible scenario: in real situations the system is always finite and as a result of many interdependent variables the system could be subject to time interplay of balanced and unbalanced mating probabilities. In this case the allele frequency time series would be typically a sequence of time periods of random drift separated by periods of stable allele frequencies in such a way that observed values of χ*2* are typically small at any time. Depending on how frequently the system jumps between the balanced and the unbalanced context (which would depend on additional dynamical rules) the properties of equilibrium states could be hardly identified. One may easily conceive a dynamical portrait where different stable and neutral equilibrium states dynamically appear and disappear leading to a situation close to the idea of metastable states that are locally (in a small interval of the allele frequencies) but not globally (for all values of the allele frequencies) stable. Therefore, the structure of the dynamics in genetic systems may be very subtle, complex, and hardly identifiable using simple tools like the χ^2^-test. As a conclusion the notion of equilibrium states, absent in Hardy (1908) original letter, deserves deeper analysis well beyond the simplistic discussions found in textbooks and papers.

**Figure 5:**
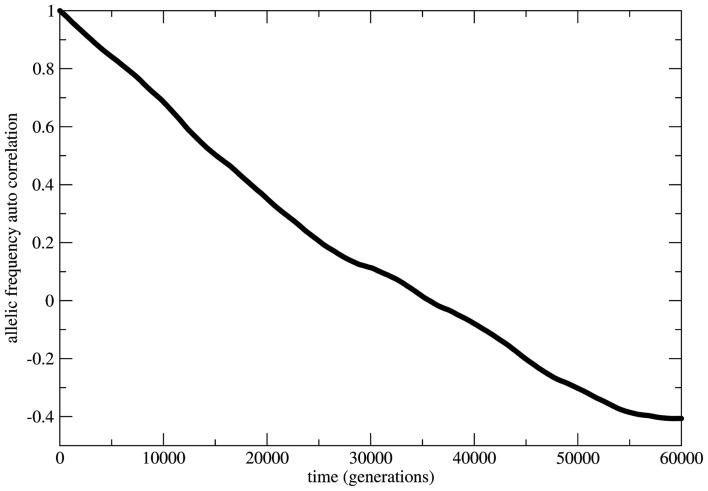
**The allelic frequency auto-correlation function for the case of random mating**. The data were obtained from the allele frequency time series for a population of 3.0 × 10^6^ individuals during a history of 3.0 × 10^5^ generations. The auto-correlation function shows that the genetic system does not reach an equilibrium state till at least 60,000 generations.

**Figure 6:**
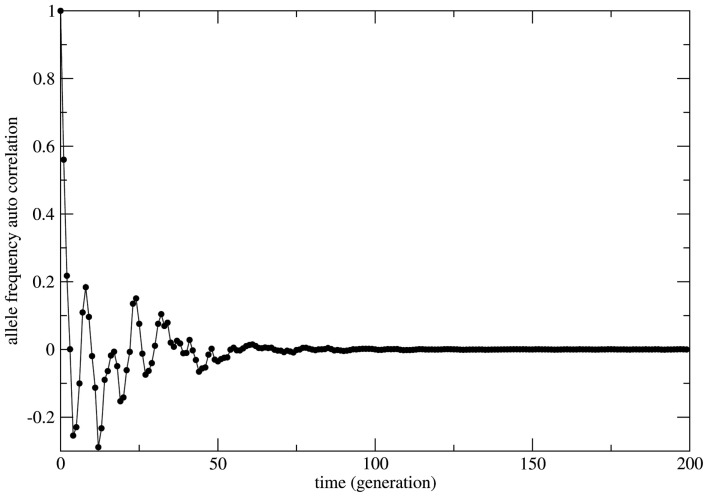
**The allelic frequency auto-correlation function for one representative case of non-random mating**. In order to show the transient regime plus the achievement of equilibrium the numerical simulation was performed with an initial population (*N*_aa_ = 10, *N*_AA_ = 10, and *N*_Aa_ = 20) that grows till a population size of 3.0 × 10^7^ individuals. The simulation total time is 3.0 × 10^5^ generations. After approximately 100 generations the correlation function is very close to zero indicating that (stable) equilibrium has been reached.

In several cases the problem of characterizing equilibrium states is very complex to be treated analytically. In these cases it is usual to have only the output time series of relevant observables as workable data and the important quantity to be examined is the time auto-correlation function of the relevant observable. For systems driven by stable equilibrium states this function presents fast (exponential like) decay to zero with a characteristic decay rate that relates to the typical time necessary to achieve the equilibrium (asymptotic) state; the time to achieve equilibrium is an intrinsic property of the system and is usually called the system’s relaxation time. In this perspective exponential decay of correlation functions is viewed as a signature of stable dynamics and an indirect way to identify the existence of stable equilibrium states. In other terms, stable dynamics leads to (fast enough) exponential decay of correlations characterizing short transients and stable equilibrium states achievable in finite time. That is the case of genetic systems subject to constant (external) unbalanced mating probabilities: the auto-correlation function for the allelic frequency (considered as the system’s observable) typically decays to zero in a exponential way: this is the process of relaxation toward equilibrium. On the other hand, in the case of random mating the decay of the allelic frequency auto-correlation is typically (slow) non-exponential characterizing a system driven by neutral dynamics. The decay in these cases is so slow that one may say the system never attains an equilibrium state. The system is not driven by an equilibrium state but its dynamics is governed by an endless transient regime. One main implication of this fact is that if the system’s auto-correlation function does not relax fast enough then the (eventual) existence of an equilibrium state in the limit of infinite time becomes useless in helping to understand and describe the system’s dynamics. The whole dynamics is contained in the transient regime and the time history of the systems is better described as a permanent transformation without asymptotic equilibrium. That is the case of systems evolving according to neutral dynamics. If the system is rigorously infinite then the observation of transient times is impossible since the system is not appropriately relaxing; there is no variation of the allele frequency from the starting point *t* = 0. On the other hand if the system is finite (even very large) the transient regime is infinite and the system never attains a state that possibly could be classified as equilibrium unless we define it as such theoretically with no way to make it unambiguously observable. Two representative examples are given in Figures 5 and 6. In Figure 5 the system evolves according to random mating and the slow decay of correlations is visible if compared with Figure 6 showing the same allele frequency time correlation function for one case of non-random mating.

In Figure 6 the decay is typically exponential and the correlation function can be written as *C*(*t*) ≈ exp(−dt) with *T* = 1/*d* the characteristic time needed to achieve the equilibrium state; in other words the correlation function decays fast enough such that the equilibrium state is observable in finite time. On the other hand in Figure 5 the decay is slower than any exponential function in such a way that the time needed to achieve equilibrium is beyond observability (in the case of the present model rigorously infinite). This observation leads to the conclusion that under the HW conditions the genetic population is permanently relaxing toward an unattainable state of equilibrium. It should be clear that after fixing one allele the population is in a state easily identifiable as stable equilibrium since small perturbations of this state would easily die out restoring the state of fixed allele.

## Discussion

The relevance of the results above described is based on addressing the fundamental dynamical aspect of the concept of equilibrium (states) in relation to the idea that under HW conditions genetic populations show time invariance of the allele frequencies. Therefore, to characterize the equilibrium states is a central issue. In order to identify the properties of the equilibrium state revealed by the system’s time series one should apply dynamical criteria and not statistical ones. Experimental studies attempting to identify the properties of equilibrium states should try to reproduce what is shown in Figures 3–6 namely: if the same or different allele proportions are obtained after introducing perturbations and/or how allele frequency time correlations decay for systems with partial mating. This would offer sound arguments for the hypothesis of equilibrium and its properties. Moreover, we note that to fully justify the hypothesis of equilibrium states one should take in full consideration the biological/physical mechanisms involved in reproduction and their impact on the system’s history. To make our conclusions more applicable a method for analysis of time series of allele frequencies is under preparation.

Analogies between theories should be grounded on clear and sound basis going as far as possible to the foundations, otherwise it is very likely that misleading conclusions will be derived from such analogies. The attempt to apply the notion of equilibrium to genetic systems from the analogous concept in mechanics is one of these cases. The notion of a genetic state of equilibrium presented in the context of the synthetic theory implies necessarily that the driving cause of genetic transformation of the system’s state is external to it in analogy to the action of external forces acting on a mechanical system through one or more of the basic physical interactions. Therefore, the analogy here is established at the level of Newton’s second law which is a definition of force. The analogy would only be complete if one is capable to derive or identify a principle analogous to the Newton’s third law which contains the physical essence of the principles governing mechanics. If we conceive such a principle applied to genetic systems (which finally would describe how genetic systems interact with their environment) we are obliged to consider the influences due to the size of the environment in respect to the system’s size as well as the specific role of what could be external and internal forces and their nature as conservative or dissipative. The distinction between these two forces is deeply related to the existence of conservation laws and time symmetry. The general difficulty to identify a version of the Action–Reaction principle for genetic systems and the strong indications that stochasticity plays a fundamental role in genetic dynamics (not only due to sampling limitations) imposes the identification of genetic driving forces as essentially dissipative. This point is supported by the fact that genetic populations are systems with varying total mass and therefore have to be considered as open physical systems.

The above arguments suggest that a better formal analogy should be made with non-equilibrium statistical physics rather than with mechanics which would focus on the search of basic principles possibly related to the second law of thermodynamics or the flux of fundamental quantities like entropy. In dealing with open systems we have the conceptual advantage that independently of the chosen driving (extreme) principle it is quite natural to expect the theory could describe the existence of stationary states that may be classified as stable (locally or globally), metastable, unstable, or even situations where a large number of different stationary states may coexist leading to very complex dynamical portraits. As it should be clear at this point, in pushing the analogy with non-equilibrium thermodynamics and statistical physics we are forced to abandon the analogy with mechanical equilibrium and consider the idea of genetic equilibrium states as stated by the HW principle just as an unattainable idealization in the same way closed isolated thermodynamic systems are idealized objects with no counterpart in reality. But here again the analogy is not complete: ideal thermodynamic systems are of great importance to put in evidence the existence of general laws governing their dynamical behavior (the laws of equilibrium thermodynamics and the emergence of dissipative structures out of equilibrium). For genetic systems this is not the case so far.

## Conflict of Interest Statement

The authors declare that the research was conducted in the absence of any commercial or financial relationships that could be construed as a potential conflict of interest.
